# Supplementary material for: The effects of genital myiasis on the diversity of the vaginal microbiota in female Bactrian camels
Source: BMC Vet Res. 2022 Mar 5;18:87. doi: 10.1186/s12917-022-03189-5 (PMC8897907; doi:10.1186/s12917-022-03189-5)
Supplement: Supplementary file 5 — Additional file 5. [file 12917_2022_3189_MOESM5_ESM.zip › MPL201709200_16s_yy/Treat1/css/js_css/pdfmake-master/examples/pdfs/absolute.pdf]

You can put images at any position

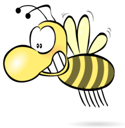

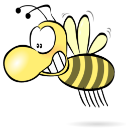

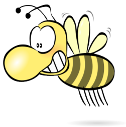

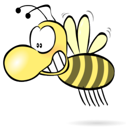

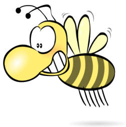

As !!!

well text

as

And this is a table on top of an image at x:100 y:100

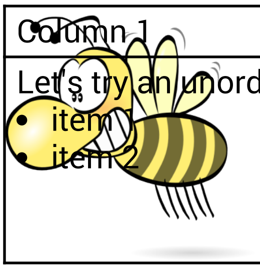

<table>
<tr><td>Column 1</td><td>Column 2</td><td>Column 3</td></tr>
<tr><td>Let's try an unordered list
• item 1
• item 2</td><td>or a nested table<table><tr><td>Col1</td><td>Col2</td><td>Col3</td></tr><tr><td>1</td><td>2</td><td>3</td></tr><tr><td>1</td><td>2</td><td>3</td></tr></table></td><td>Inlines can be styled
easily as everywhere else</td></tr>
</table>
